# CD103^+^ Conventional Dendritic Cells Are Critical for TLR7/9-Dependent Host Defense against *Histoplasma capsulatum*, an Endemic Fungal Pathogen of Humans

**DOI:** 10.1371/journal.ppat.1005749

**Published:** 2016-07-26

**Authors:** Nancy Van Prooyen, C. Allen Henderson, Davina Hocking Murray, Anita Sil

**Affiliations:** 1 Department of Microbiology and Immunology, University of California San Francisco, San Francisco, California, United States of America; 2 Howard Hughes Medical Institute, San Francisco, California, United States of America; University of Wisconsin-Madison, UNITED STATES

## Abstract

Innate immune cells shape the host response to microbial pathogens. Here we elucidate critical differences in the molecular response of macrophages vs. dendritic cells (DCs) to *Histoplasma capsulatum*, an intracellular fungal pathogen of humans. It has long been known that macrophages are permissive for *Histoplasma* growth and succumb to infection, whereas DCs restrict fungal growth and survive infection. We used murine macrophages and DCs to identify host pathways that influence fungal proliferation and host-cell viability. Transcriptional profiling experiments revealed that DCs produced a strong Type I interferon (IFN-I) response to infection with *Histoplasma* yeasts. Toll-like receptors 7 and 9 (TLR7/9), which recognize nucleic acids, were required for IFN-I production and restriction of fungal growth in DCs, but mutation of TLR7/9 had no effect on the outcome of macrophage infection. Moreover, TLR7/9 were essential for the ability of infected DCs to elicit production of the critical cytokine IFNγ from primed CD4^+^ T cells *in vitro*, indicating the role of this pathway in T cell activation. In a mouse model of infection, TLR7/9 were required for optimal production of IFN-I and IFNγ, host survival, and restriction of cerebral fungal burden. These data demonstrate the critical role of this pathway in eliciting an appropriate adaptive immune response in the host. Finally, although other fungal pathogens have been shown to elicit IFN-I in mouse models, the specific host cell responsible for producing IFN-I has not been elucidated. We found that CD103^+^ conventional DCs were the major producer of IFN-I in the lungs of wild-type mice infected with *Histoplasma*. Mice deficient in this DC subtype displayed reduced IFN-I production in vivo. These data reveal a previously unknown role for CD103^+^ conventional DCs and uncover the pivotal function of these cells in modulating the host immune response to endemic fungi.

## Author Summary

Innate immune cells such as macrophages and dendritic cells (DCs) are critical elements of the initial response to pathogens. Whereas both of these cell types utilize robust anti-microbial strategies to kill internalized microbes, intracellular pathogens have developed mechanisms to manipulate the host response and survive within host cells. In the case of the intracellular fungal pathogen *Histoplasma capsulatum*, the fungus proliferates within macrophages, resulting in host-cell lysis. In contrast, DCs are able to restrict *Histoplasma* growth. Here we discovered that the ability of DCs to produce Type I interferons (IFN-I) is critical to their capacity to restrict fungal proliferation and survive infection. IFN-I are cytokines that are elicited during a variety of viral, bacterial, and fungal infections. We performed in vivo and in vitro experiments to show that pattern recognition receptors TLR7 and TLR9 are critical for the IFN-I response and host survival in the mouse model of infection. Additionally we defined a specific DC subset (CD103^+^ conventional DCs) in the mouse lung that is responsible for the IFN-I response, revealing a previously unknown role for these cells. These data provide insight on the pivotal role of a specific host-response pathway at both a cellular and organismal level during infection with endemic fungi.

## Introduction

Key functions of the innate immune system include pathogen recognition, effector cytokine production, and orchestration of an adaptive immune response. Type I interferons (IFN-I) are key effector cytokines that are produced by a variety of innate immune cells. In both humans and mice, the IFN-I family is comprised of 13 IFN-α species, a single IFN-β, and other less-studied members (IFN-ω, -ε, -κ) [1, 2]. The initiation of a proper IFN response plays a critical role in antimicrobial clearance by limiting the spread of infection and orchestrating the initial phases of the adaptive immune response. However, the mechanism of detection and IFN production varies greatly depending on the pathogen and site of infection [1, 3]. Much of our information about the IFN-I response comes from viral and bacterial infection models, whereas the mechanism of induction in response to a fungal pathogen remains relatively unexplored [4].

IFN-I production by innate immune cells can be triggered by a number of distinct pathways. For example, pathogens that present nucleic acids in the phagosome can trigger recognition by phagosomal Toll-like receptors (TLRs), including TLR3, TLR7, and TLR9 [5–9]. TLR3 responds to double-stranded RNA, a replication intermediate for many viruses, and signals through the downstream adapter TRIF. TLR7 recognizes single stranded RNAs whereas TLR9 recognizes unmethylated CpG motifs, and both of these TLRs signal through the downstream adapter MyD88. As a result, several transcription factors including NF-κB and interferon regulatory factors (IRFs) are activated, leading to IFN-I production. In contrast, other pathogens that access the cytosol can trigger a cytosolic surveillance pathway that results in IFN-I production independent of MyD88 and TRIF [10].

Although various cells are reported to have the potential to produce IFN-I in vivo, the main contributors are thought to be dendritic cells (DCs), which are potent antigen presenting cells that undergo maturation after exposure to inflammatory stimuli [11]. Mature DCs migrate from the peripheral tissues into the T cell areas of secondary lymphoid organs, where they present antigens and initiate a T cell response. In the mouse lung, plasmacytoid DCs (pDCs), CD11b^+^ conventional DCs (cDCs) and CD103^+^ cDCs comprise the three main DC subclasses [12]. pDCs are known to produce high levels of IFN-I upon exposure to several viruses and bacteria [13], but little is known about the role of different DCs subtypes in the context of a fungal infection.

Here we focus on how cells of the innate immune system respond to the fungal pathogen *Histoplasma capsulatum* and the role of IFN-I in this host-pathogen interaction. *Histoplasma* is a primary fungal pathogen that infects immuncompetent individuals, and its ability to subvert the innate immune response of a healthy host makes it an excellent model to investigate the mammalian immune response to pathogens. In endemic regions (in the Midwestern United States and other parts of the world), *Histoplasma* grows in soil as a filamentous mold that produces fungal spores known as conidia. Conidia and fragments of filaments aerosolize and are inhaled by a mammalian host. Once inside the host, fungal cells switch their growth program to give rise to unicellular pathogenic yeast that reside within phagocytic innate immune cells. The intracellular fate of *Histoplasma* depends on the type of phagocytotic innate immune cell that engulfs it. Whereas *Histoplasma* safely resides and replicates within macrophages, DCs are able to restrict *Histoplasma* growth [14]. Neutrophils can also phagocytose *Histoplasma* [15, 16], but their role in infection remains poorly understood. Ultimately fungal cells are thought to use cells of the reticuloendothelial system to disseminate from the lung to multiple body sites [17, 18].

We previously determined that macrophages do not produce IFN-I in response to infection with *Histoplasma* yeast cells [19]. In the present study, we show that *Histoplasma* yeasts induced a strong IFN-I response in cultured DCs in a TLR7/9-dependent manner, and that this IFN-I response was required for the ability of DCs to restrict intracellular fungal growth and survive fungal infection. In the mouse, TLR7/9 was required for IFN-I production in response to *Histoplasma* infection, and mice lacking TLR7/9 experienced higher fungal burden, increased neutrophil recruitment, increased lung damage, colonization of the brain by *Histoplasma*, and increased mortality. Furthermore, TLR7/9 were necessary for appropriate DC and CD4^+^ T cell interactions that promote T cell activation and cytokine production. Surprisingly, the main IFN-I-producing cells in infected lungs were the CD103^+^ cDCs, indicating a previously unappreciated role for these cells in a protective host response to an endemic fungal pathogen. Our study highlights the importance of IFN-I production by CD103^+^ cDCs for pathogen restriction and host survival during infection with a ubiquitous pathogen of humans.

## Results

### *Histoplasma-*infected DCs trigger an IFN-I transcriptional response that is critical for fungal restriction and host-cell survival

A number of studies have shown that the fate of *Histoplasma* within host cells differs in macrophages and DCs [20, 21]. To analyze the outcome of infection in murine macrophages and DCs, we differentiated murine bone marrow into either macrophages (bone marrow macrophages, BMMs) or dendritic cells (bone marrow dendritic cells, BMDCs) and co-cultured these cells with *Histoplasma* yeasts. In a recent study, BMDCs were shown to be a heterogeneous population of cDCs and monocyte macrophages [22] depending on the differentiation procedure. We generated BMDC used in this study by differentiating bone marrow with mIL4 and mGM-CSF for seven days, followed by a CD11c column purification. Flow cytometry analysis revealed that a small population of the cells were monocyte macrophages, but the majority of cells (70–80%) were CD11c^+^CD11b^+^MHCII^+^CD135^+^. In addition, these cells did not express CD103, suggesting that our BMDCs resembled CD11b^+^ cDCs and not CD103^+^ cDCs. Less than 10% of these cells expressed markers (B220 and SiglecH) used to identify pDCs (S1 Fig) [22].

**Fig 1:**
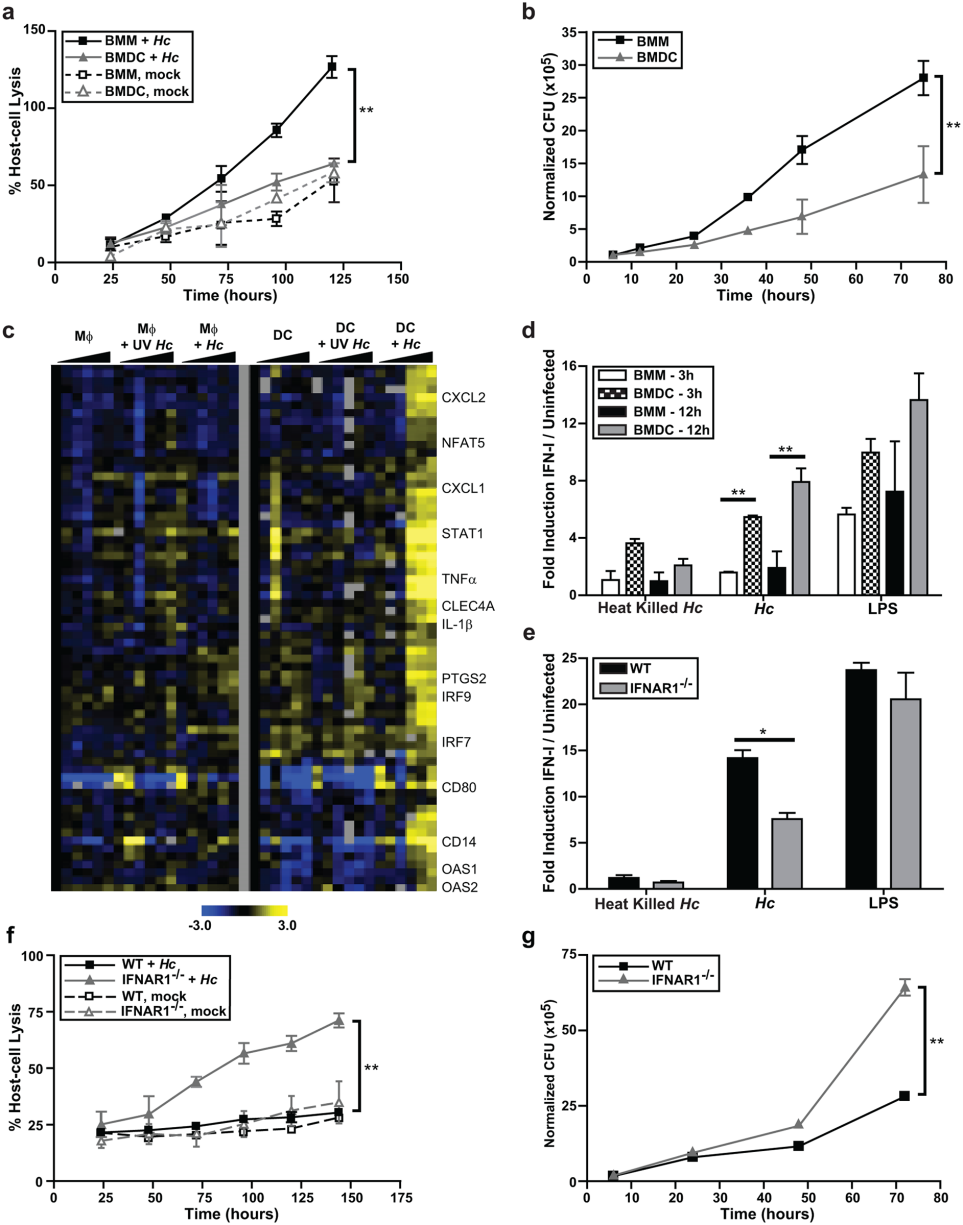
Dendritic cells induce an IFN-I response during infection with *Histoplasma* yeasts. BMMs or BMDCs were either mock-infected or infected with live *Histoplasma capsulatum* (*Hc*) yeasts at an MOI of 2. **(A)** Lactate dehydrogenase (LDH) was measured to monitor host cell lysis. The average percentage lysis of four measurements and representative experiment of 3 replicates is shown. **(B)** Host cells (BMMs or BMDCs) were washed then osmotically lysed and colony-forming units (CFUs) representing intracellular yeast cells were enumerated. A representative experiment of 3 replicates is shown. **(C)** BMMs or BMDCs were cocultured with either UV-treated (UV) or live yeast at an MOI of 4. Gene expression was examined by microarray analysis. Each set of columns under the black triangle corresponds to the following time points: 1, 3, 6, 12, 24, and 36 hpi. The cluster displayed was based on genes upregulated in BMDCs infected with live *Histoplasma*. Yellow indicates an increase in gene expression relative to mock-infected samples, blue indicates a decrease, black indicates no change, and gray indicates missing data. **(D)** BMMs or BMDCs were either treated DMSO carrier or LPS (10ng/ml), and cocultured with heat killed or live *Histoplasma* at an MOI of 4. Supernatants were collected at either 3 hpi or 12 hpi. The amount of IFN-I in the media was detected by using a reporter cell line, ISRE-L929. A representative experiment of 3 independent replicates is shown. **(E, F, G)** BMDCs differentiated from C57Bl/6J (WT) or IFNAR1-/- mice bone marrow were cocultured with either heat-killed or live *Histoplasma*. Data representative of 2 experiments is shown. **(E)** IFN-I protein in the medium was measured at 12 hpi with ISRE-L929 IFN-I reporter cells. (**F**) Host-cell lysis was measured by LDH release and **(G)** CFUs were numerated. All error bars indicate SD (standard deviation). *p<0.05; **p<0.001; p values were determined by ANOVA.

Here we show BMDCs had considerably less lysis and greater fungal restriction than BMMs (Fig 1A and 1B). To shed light on the differential responses of these host cells, we used Mouse Exonic Evidence-Based Oligonucleotide (MEEBO) microarrays (Illumina) to determine their transcriptional profiles during infection. BMMs and BMDCs were cocultured with either UV-treated or live *Histoplasma* yeasts. Samples were collected to enumerate fungal burden (S2 Fig) and generate host RNA at multiple time points. We reproduced the previously reported the gene expression response of BMMs to *Histoplasma* [23], but in this analysis we were particularly interested in genes that were upregulated by BMDCs but not BMMs. We discovered that a cluster comprised of IFN-I responsive genes was highly induced only in response to live *Histoplasma* yeast in BMDCs at 12 to 36 hours post-infection (hpi) (Fig 1C). Consistent with our previous report [19], BMMs did not induce IFN-I genes in response to *Histoplasma* yeasts (Fig 1C).

Our transcriptional data suggested that host cells produce IFN-I protein in response to *Histoplasma* infection. We used an interferon reporter mouse fibroblast cell line (ISRE-L929, which produces luciferase in response to all IFN-I) as a sensitive assay to detect Type I IFN proteins in the supernatants of infected cells [24, 25]. BMMs and BMDCs were cocultured with either heat-killed or live *Histoplasma*, and culture supernatants were collected and added to ISRE-L929 cells for 5–8 hours. Whereas both BMMs and BMDCs produced IFN-I protein in response to LPS (which was used as a positive control), only BMDCs were able to produce IFN-I in response to live *Histoplasma* yeasts (Fig 1D). A low magnitude response was observed in BMDCs cocultured with heat-killed *Histoplasma* at 3 hpi, which declined in amplitude by 12 hpi (Fig 1D). These data indicate that live *Histoplasma* yeast trigger a strong IFN-I response in DCs.

To determine whether IFN-I contributes to the survival of BMDCs during *Histoplasma* infection, we cocultured wild-type BMDCs or BMDCs lacking the IFNAR1 subunit of the Type I IFN receptor IFNAR (IFNAR1^-/-^) with either live or heat-killed *Histoplasma*. After release of IFN-I from producing cells, IFN-I binds to IFNAR, thereby triggering a secondary gene expression response that includes the induction of interferon-stimulated genes and the transcriptional induction of more IFN-I [26]. As expected, IFNAR^-/-^ DCs showed a significant reduction in IFN-I protein production (Fig 1E). Notably, IFNAR^-/-^ DCs were considerably more susceptible to host-cell lysis during *Histoplasma* infection (Fig 1F), and were defective in restriction of fungal growth (Fig 1G). Taken together, these data indicate that IFN-I-induced genes contribute to the ability of DCs to control *Histoplasma* infection.

### Dendritic cell IFN-I production is dependent on MyD88 and TLR7/9

**Fig 2:**
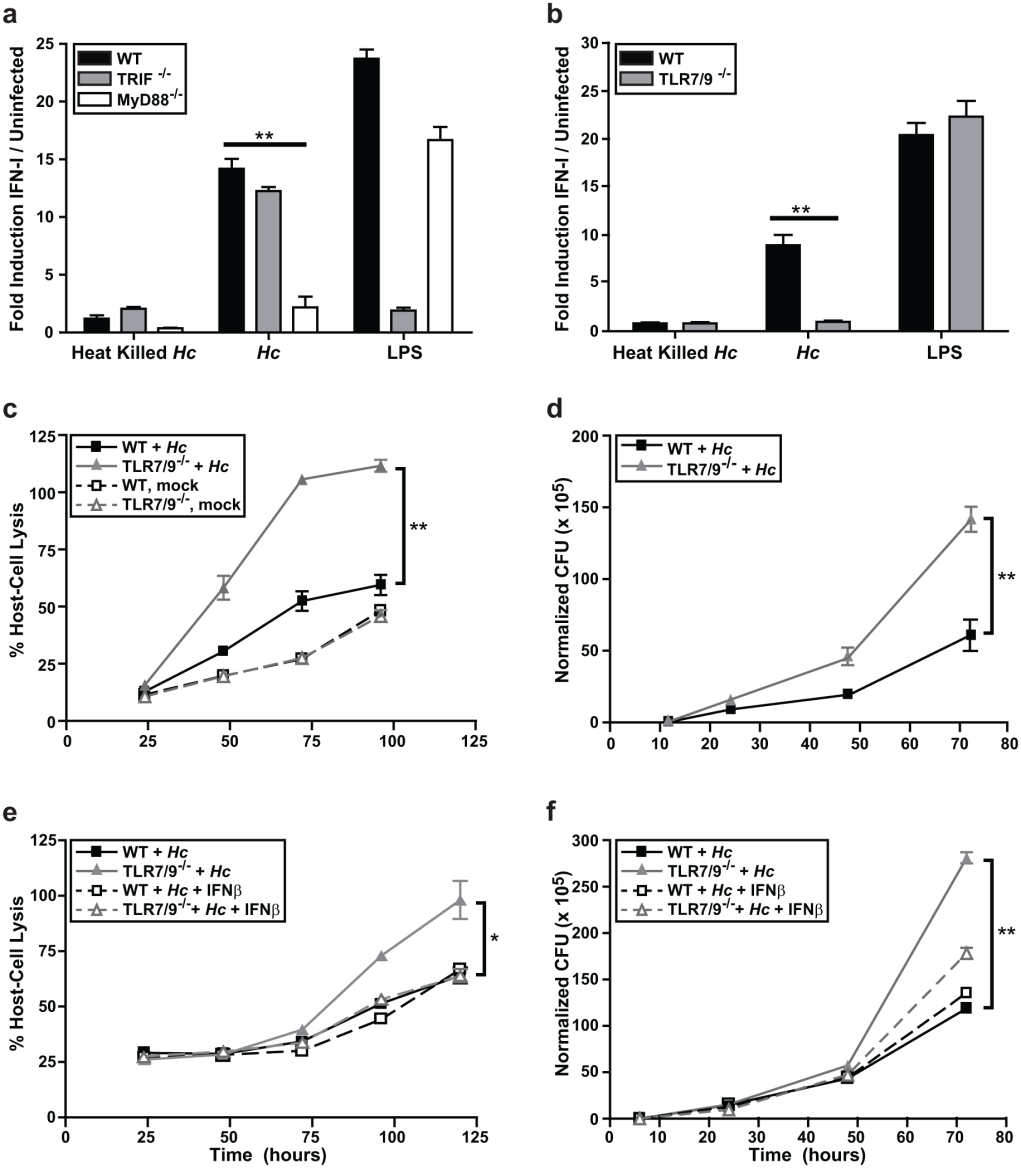
IFN-I production and *Histoplasma* restriction in DCs is dependent on MyD88, TLR7, and TLR9 signaling. **(A, B)** WT, TRIF^-/-^, MyD88^-/-^, or TLR7/9^-/-^ BMDCs were cocultured with heat-killed or live *Histoplasma capsulatum* (*Hc*) yeasts at an MOI of 4. As a positive control, BMDCs were treated with LPS. IFN-I was measured at 12 hpi using the ISRE reporter line. **(C-F)** wild-type or TLR7/9^-/-^ BMDCs were either mock-infected or infected with *Histoplasma* at an MOI of 2. **(C)** Infections were monitored for host cell lysis via LDH activity. **(D)** CFUs from infections were enumerated. **(E, F)** BMDCs were either treated with control vehicle or mouse recombinant IFNβ. Cells post-infection were monitored for **(E)** host cell lysis via LDH activity and **(F)** CFUs. All experiments are representative of 3 independent experiments and error bars indicate SD. **p<0.001; p values were determined by ANOVA.

Given the importance of the IFN-I pathway in the dendritic cell response to *Histoplasma*, we next focused on understanding which host pathways are required to induce this response. The IFN-I response in immune cells is induced in response to either phagosomal or cytosolic signaling pathways. Since *Histoplasma* is a vacuolar pathogen, we asked whether yeast cells induced IFN-I in response to phagosomal pattern recognition receptors TLR3, TLR7, and TLR9. Since TLR7 and TLR9 signal through the adaptor protein MyD88 and TLR3 signals through the adaptor protein TRIF [27, 28], we first tested whether IFN-I protein production in BMDCs was dependent on either MyD88 or TRIF. We measured IFN-I protein production in BMDCs differentiated from WT, MyD88^-/-^, or TRIF^-/-^ bone marrow cocultured with either live or heat-killed *Histoplasma* yeast. The IFN-I response in BMDCs was independent of TRIF, but dependent on MyD88 (Fig 2A). Consistent with this observation, we recently reported that MyD88 is critical for control of *Histoplasma* infection in the mouse model of infection [29].

Since phagosomal TLR7 and TLR9 signal through MyD88 to stimulate IFN-I production, we next tested whether these receptors are required for the IFN-I response of BMDCs. BMDCs prepared from TLR7^-/-^ or TLR9^-/-^ bone marrow showed a significantly reduced level of IFN-I production (S3 Fig), and BMDCs prepared from TLR7/9^-/-^ double knockout bone marrow were completely defective in the IFN-I response to *Histoplasma* (Fig 2B). Notably, TLR7 and TLR9 were essential for the ability of BMDCs to survive *Histoplasma* infection (Fig 2C, S3B and S3D Fig) and restrict fungal growth (Fig 2D, S3C and S3E Fig). Interestingly, the addition of exogenous recombinant IFN-β (50U/ml) to TLR7/9^-/-^ BMDCs during *Histoplasma* infection significantly decreased the level of host cell lysis (Fig 2E) and intracellular *Histoplasma* growth (Fig 2F). Importantly, TLR7/9^-/-^ BMDCs treated with IFN-β resembled wild-type BMDCs in terms of their ability to control *Histoplasma* infection. These data highlight the critical role of IFN-I in the ability of BMDCs to restrict *Histoplasma* growth and protect host cells from lysis.

### TLR7/9 has a protective role in the mouse model of *Histoplasma* infection

**Fig 3:**
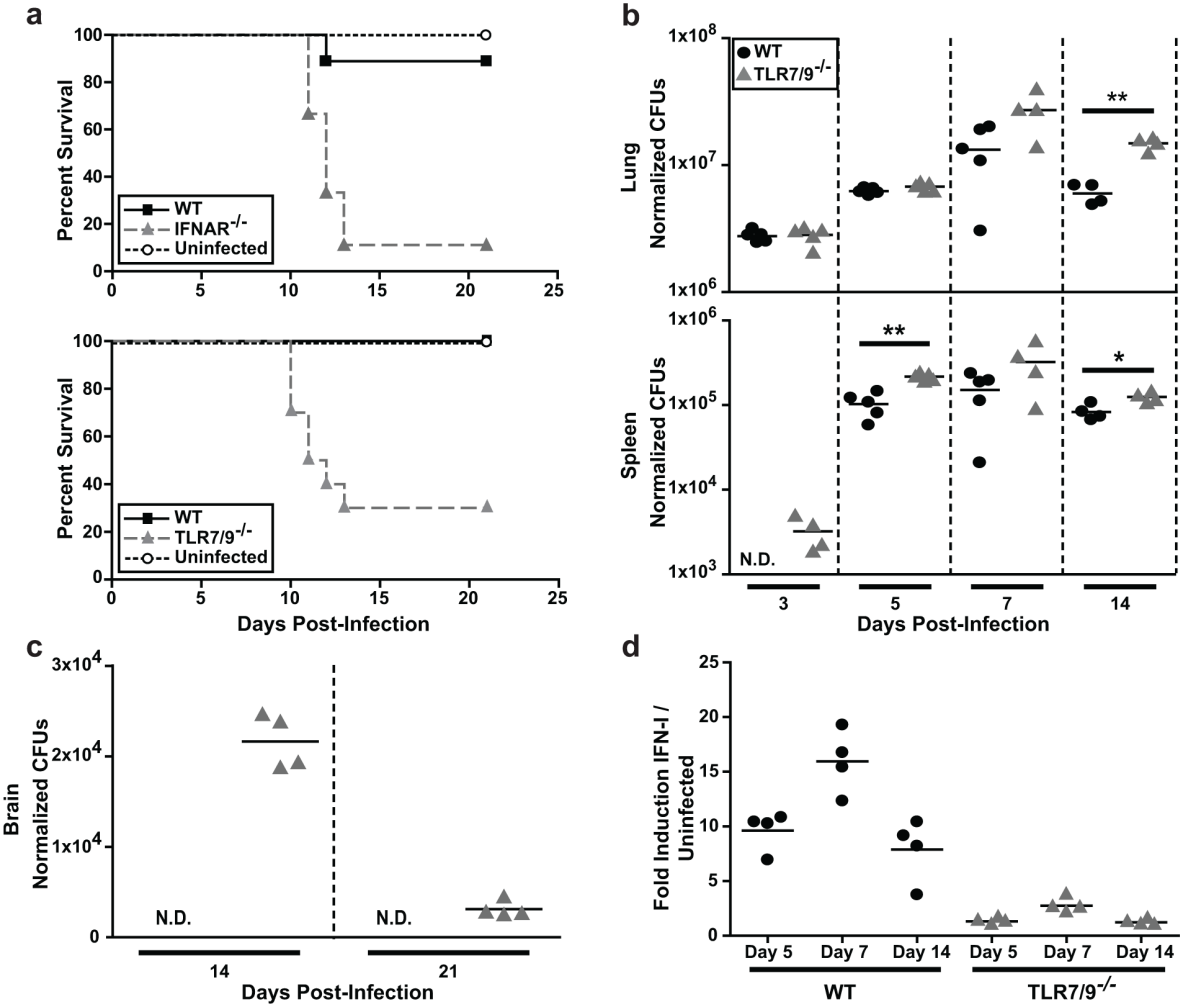
TLR7/9 are critical for IFN-I production and host survival in the mouse model of *Histoplasma* infection. Mice were intranasally infected with a sub-lethal dose of 3x10^5^ *Histoplasma* yeasts. **(A)** Kaplan-Meir survival curves of female wild-type (n = 10), IFNAR^-/-^ (n = 10), TLR7/9^-/-^ (n = 10) or PBS-treated (uninfected) (n = 4) mice. **(B)** Lungs, spleens and **(C)** brains of infected wild-type and TLR7/9^-/-^ mice were harvested, homogenized and plated for CFUs at the indicated days post-infection (dpi) (n = 5 mice/time-point). **(D)** IFN-I was measured in wild-type and TLR7/9^-/-^ lung homogenates using ISRE reporter cells. Each symbol represents a single mouse. All results are representative of at least three experiments. *p<0.05; **p<0.001; p values were determined by ANOVA.

We previously observed that IFNAR^-/-^ mice infected with 2x10^4^ *Histoplasma* yeast cells had a decreased pulmonary fungal burden at 14 dpi compared to wild-type mice [19]. However, in that study we did not determine survival differences between wild-type and IFNAR^-/-^ mice infected with *Histoplasma* yeast cells. Thus to further elucidate the significance of the IFN-I response in host defense against *Histoplasma* infection, we infected WT, IFNAR^-/-^, or TLR7/9^-/-^ mice intranasally with a sub-lethal dose of 5x10^5^ yeast cells. The majority (70–90%) of IFNAR^-/-^ and TLR7/9^-/-^ mice succumbed by 14 days post-infection (dpi), whereas 90–100% of the wild-type mice survived the course of the experiment (Fig 3A). TLR7/9^-/-^ mice showed significantly increased fungal burden in the lungs by 10 dpi (S4 Fig), which corresponds to when the majority of these mice begin to succumb to infection. Additionally, *Histoplasma* was not detected in the spleens of wild-type mice at 3 dpi, whereas it did accumulate in the spleens of TLR7/9^-/-^ mice by this early time point (Fig 3B).

The small percentage of TLR7/9^-/-^ mice that did not succumb to infection by 14 dpi displayed unusual behavior, such as rapid movements, walking backwards, and running into the sides of the cage. These observations prompted us to examine whether these mice had fungal burden in their brains. Unlike wild-type mice, TLR7/9^-/-^ mice had detectable levels of *Histoplasma* in the brain at 10 dpi (S4A Fig) and a peak fungal burden in the brain at 14 dpi (Fig 3C). Since brain colonization has not been extensively characterized in the mouse model of histoplasmosis, we infected TLR7/9^-/-^ mice with mCherry-producing *Histoplasma* and performed flow cytometry analysis on brain homogenates at the time of peak colonization (14 dpi). We observed that the majority of *Histoplasma* was extracellular (~55%) whereas the remainder of yeast cells were found within microglia (~35%) or DCs (~5%) (S4B Fig). *Histoplasma* was also visualized in brain sections (S4C Fig).

Finally, to assess whether TLR7/9^-/-^ mice were deficient in IFN-I production, we exposed ISRE-L929 IFN-I reporter cells to lung homogenates from infected mice. We observed a peak of IFN-I production at 7 dpi in wild-type mice and a significant reduction in IFN-I from TLR7/9^-/-^ mice at all time points (Fig 3D), indicating that TLR7/9 are required for IFN-I production in the lungs of infected mice.

### TLR7/9 are required for IFN-I production by lung CD103^+^ cDCs

**Fig 4:**
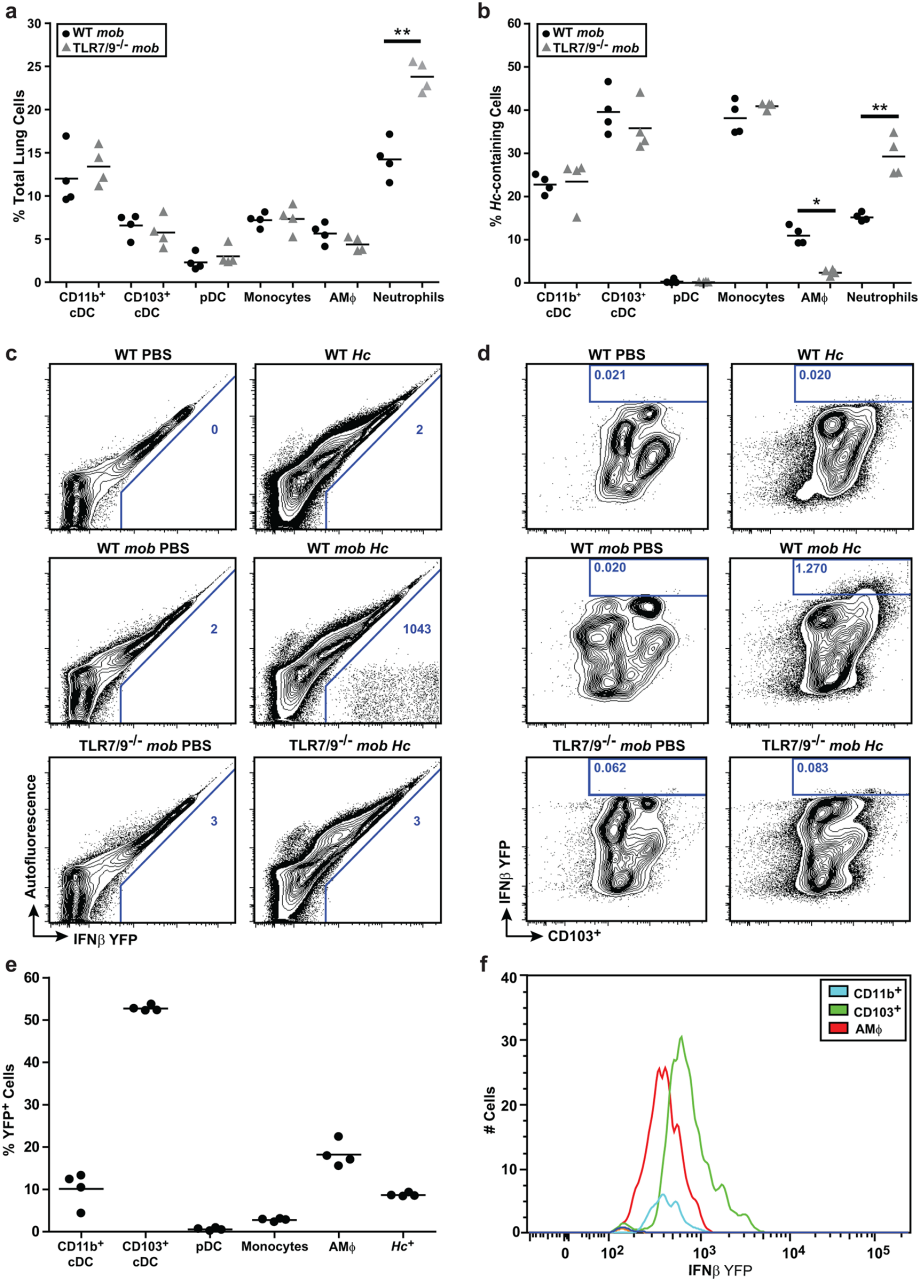
CD103^+^ cDCs and alveolar macrophages produce IFN-I in the lung during *Histoplasma* infection. wild-type *mob* and TLR7/9^-/-^ *mob* reporter mice were infected with 3x10^5^ mCherry-*Hc* yeast cells. **(A)** Lungs 7 dpi were analyzed by flow cytometry for cell surface markers to identify immune cells (CD11b^+^ cDCs, CD103^+^cDCs, pDCs, alveolar macrophages (AMΦ) and neutrophils) **(B)** the percentage of mCherry-*Hc*-containing cells **(C-F)** and IFNβ/YFP expression. **(C)** The number represented in each box is the frequency of YFP^+^ cells per million lung cells. **(D)** The box represents the total population of CD103^+^ cDCs that are IFNβ/YFP positive, which were gated as CD45^+^MHCII^+^CD11c^+^CD24^+^. The number represented in each box is the percentage of positive IFNβ/YFP CD103^+^ cDCs in the total CD45^+^ population. **(E)** Analysis of IFNβ/YFP positive cells of *Hc*-infected wild-type mice and percentage of cell types that comprise this population. **(F)** Cell number vs. Mean Fluorescence Intensity of IFNβ/YFP-positive cells from *Hc*-infected wild-type mice. Each symbol represents a single mouse. Cells were defined by the following markers: neutrophils (CD11c^-^CD11b^+^SiglecF^lo^Ly6G^+^), monocytes (CD11c^-^CD11b^+^MHCII^-^CD64^+^), CD103^+^ cDCs (MHCII^+^CD11c^+^CD11b^-^CD24^+^CD103^+^), CD11b^+^ cDCs (MHCII^+^CD11c^+^CD11b^+^CD103^-^), and pDCs (CD11c^+/-^CD11b^-^CD103^-^B220^+^). All results are representative of at least three experiments. *p<0.05; **p<0.001; p values were determined by ANOVA.

In our *in vitro* experiments, BMDCs but not BMMs produced IFN-I in response to *Histoplasma* infection. To determine which cell populations produce IFNβ in vivo, we infected IFNβ/YFP reporter mice (messenger of beta interferon (*mob*) mice [30]) with a sub-lethal dose of mCherry^+^ *Histoplasma*. Additionally, we infected *mob* reporter mice that lacked both TLR7 and TLR9 (TLR7/9^-/-^ *mob* mice). We examined host cells at 7 dpi since this time corresponds to the peak IFN-1 production in lungs of wild-type mice and is also earlier than 10 dpi when TLR7/9^-/-^ mice start to succumb to infection. We used flow cytometry to determine the cellular population of monocytes, CD11b^+^ cDCs, CD103^+^ cDCs, pDCs, alveolar macrophages, and neutrophils in the lungs (S5 Fig). We observed equal numbers of total CD11b^+^ cDCs, CD103^+^ cDCs, pDCs, and alveolar macrophages in the lungs of both wild-type and TLR7/9^-/-^ mice. However, the TLR7/9^-/-^ mice showed a significant increase in lung neutrophils (Fig 4A), which comprised the majority of mCherry^+^ *Histoplasma*-containing host cells in the TLR7/9^-/-^ mice (Fig 4B). Interestingly, the TLR7/9^-/-^ mice showed a significant decrease in alveolar macrophages containing mCherry^+^ *Histoplasma* (Fig 4B).

IFNβ/YFP producing cells were observed in *mob* mice infected with *Histoplasma*, but not in *mob*-TLR7/9^-/-^ mice (Fig 4C). The majority (~52%) of IFNβ/YFP producing cells in *mob* mice were CD103^+^ cDCs (Fig 4D and 4E) whereas the remainder (<20%) were alveolar macrophages and CD11b^+^ cDCs (Fig 4E). Notably, the CD103^+^ cDCs displayed the strongest IFNβ/YFP signal per cell (Fig 4F), indicating that the CD103^+^ cDCs were producing higher levels of IFNβ than macrophages. Interestingly, only ~10% of IFNβ/YFP cells contained mCherry-*Hc* (Fig 4E, last column), suggesting that the majority of IFNβ-producing cells at 7 dpi were either bystander cells or cells that had already cleared intracellular *Histoplasma* infection.

**Fig 5:**
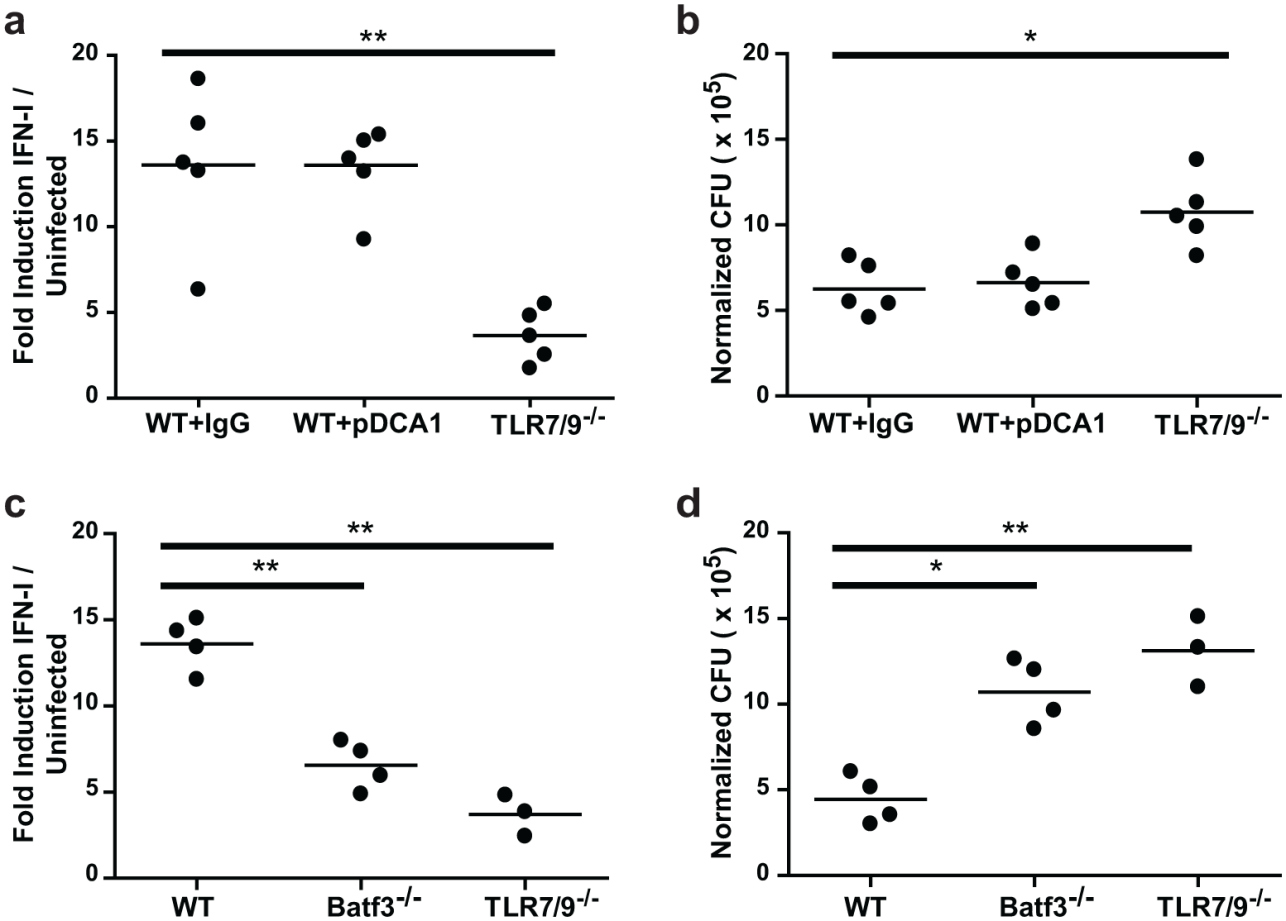
CD103^+^ cDCs are the main IFN-I producing cells in response to *Histoplasma* infection. **(A, B)** wild-type mice were treated with either rat IgG (control) or α-pDCA1 (pDC depletion) one day before infection and then at 1, 3, 5, 6 dpi. Mice were either mock-infected or infected with 3x10^5^ *Histoplasma* yeasts. **(C, D)** WT, Batf3^-/-^, or TLR7/9^-/-^ mice were infected with 3x10^5^ *Histoplasma* cells. Lungs were collected at 7 dpi and **(A, C)** total IFN-I was measured with ISRE reporter cells and **(B, D)** CFUs were enumerated. Each circle represents a single mouse. All results are representative of two experiments. *p<0.05; **p<0.001; p values were determined by ANOVA.

Since pDCs are a major source of IFN-I during viral and bacterial infections [31], it was surprising that we detected no IFNβ production from pDCs in *Histoplasma*-infected lungs. To confirm that CD103^+^ cDCs but not pDCs were required for IFN-I production we took a dual approach. First we used IP injection of the monoclonal antibody pDCA-1 to deplete pDCs before and during *Histoplasma* infection. At 7 dpi, flow cytometric analysis of spleens revealed 85–90% depletion of pDCs. The IFN-I response (Fig 5A) and fungal burden (Fig 5B) in the lungs of these mice did not differ from control mice, whereas TLR7/9^-/-^ mice infected and assessed in parallel displayed a diminished IFN-I response (Fig 5A). In a second, complementary approach, we assessed the ability of Batf3^-/-^ mice, which lack the transcription factor required for differentiation of CD103^+^ cDCs [32], to induce IFN-I and restrict *Histoplasma* infection. In contrast to wild-type mice, Batf3^-/-^ mice showed a significant decrease in IFN-I levels in the lung (Fig 5C) and a significant increase in fungal burden (Fig 5D). In comparison, TLR7/9^-/-^ mice showed an additional decrease in IFN-I levels and increase in fungal burden, indicating a second source of IFN-I independent of Batf3 (such as alveolar macrophages or CD11b^+^ cDCs, Fig 4E). Taken together, these data indicate that CD103^+^ cDCs are the major source of IFN-I during *Histoplasma* infection.

### TLR7/9 are required for normal cytokine production, activation of T cells, and IFN-γ production

**Fig 6:**
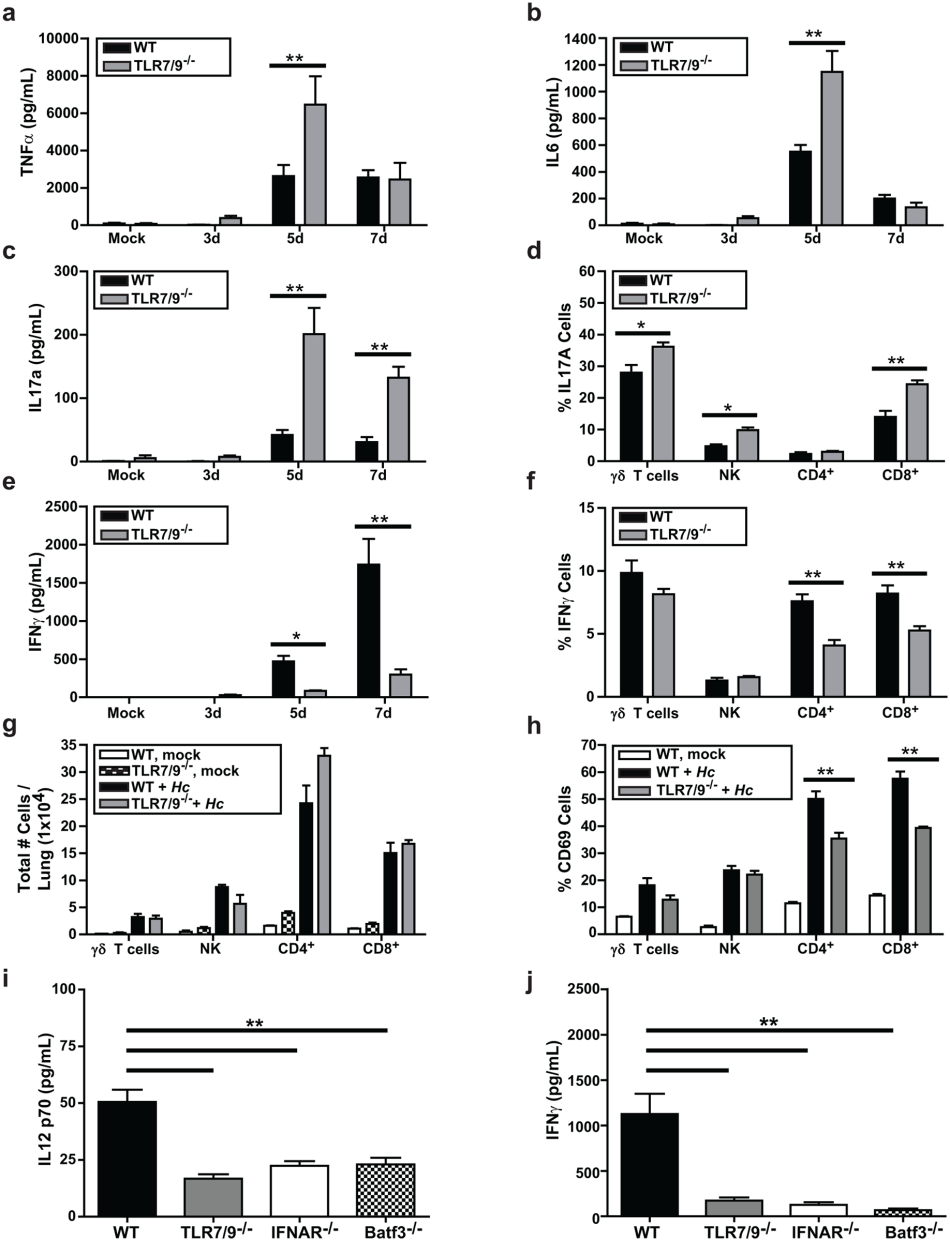
TLR7/9 is required to stimulate optimal levels of IFNγ and restrict IL17 production. Lungs of mock-infected or infected wild-type and TLR7/9^-/-^ mice were harvested, homogenized, and evaluated for cytokine levels **(A)** TNFα, **(B)** IL6, **(C)** IL17a, and **(E)** IFN-γ. **(D, F-H)** Intracellular cytokine staining on lung cells was measured to detect IFN-γ and IL17A production in γδ T cells, NK cells, CD4^+^ and CD8^+^ T cells isolated from the lung. Cells were defined as: CD4^+^ T cells (CD45^+^CD3ε^+^CD4^+^), CD8^+^ T cells (CD45^+^CD3ε^+^CD8^+^), γδ T cells (CD45^+^CD3ε^+^γδTCR^+^), NK cells (CD45^+^CD3ε^-^NK1.1^+^). **(G)** Total number of T cells per lung and **(H)** the percentage of T cells that were CD69^+^ were calculated. (**I-J**) WT, TLR7/9^-/-^, IFNAR^-/-^, and Batf3^-/-^ mice were either mock-infected or infected with 3x10^5^ *Histoplasma* yeasts. Lungs were harvested, homogenized, and evaluated for levels of **(I)** IL12 p70 and **(J)** IFNγ. All error bars indicate SD. *p < 0.05; **p < 0.001; p values were determined by ANOVA.

Since TLR7/9^-/-^ mice showed heightened susceptibility to *Histoplasma* infection, we reasoned that the host immune response to *Histoplasma* infection might be altered in these mice compared to wild-type mice. Analysis of cytokines in homogenates from infected lungs revealed that levels of pro-inflammatory cytokines TNFα and IL6 were significantly higher in the lungs of TLR7/9^-/-^ mice at 5 dpi but were equivalent to wild-type mice by 7 dpi (Fig 6A and 6B). We also examined IL17A accumulation in infected lungs and found that it was significantly increased in the TLR7/9^-/-^ mice compared to wild-type (Fig 6C). Given the role of IL17 in neutrophil recruitment [33], increased levels of IL17 may influence the abnormally high influx of neutrophils observed at 7 dpi in TLR7/9^-/-^ mouse lungs (Fig 4A). We observed that γδ T cells, NK cells, and CD8^+^ T cells all produced increased levels of IL17A in TLR7/9^-/-^ mice compared to wild-type mice. Interestingly, IL17A production by CD4^+^ T cells remained unchanged (Fig 6D).

Host survival during *Histoplasma* infection is dependent on production of IL12 and IFNγ [34, 35]. Accordingly, we examined the level of IFNγ, and found it was strongly dependent on TLR7/9 (Fig 6E). Both CD4^+^ and CD8^+^ T cells showed a dependence on TLR7/9 for maximal IFNγ induction (Fig 6F). Interestingly, although the lungs of infected wild-type and TLR7/9^-/-^ mice contained roughly equivalent numbers of NK cells, γδ T cells, CD4^+^ T-cells, and CD8^+^ T cells (Fig 6G), we saw that CD69 expression was significantly decreased in CD4^+^ and CD8^+^ T cells from TLR7/9^-/-^ mice (Fig 6H), consistent with a defect in T-cell activation (Fig 6H). Additionally, we observed that IL12 p70 production in the lung is decreased in TLR7/9^-/-^, Batf3^-/-^, and IFNAR^-/-^ mice compared to wild-type mice at 7 dpi (Fig 6I). Similarly, IFNγ levels were dependent on Batf3 and IFNAR at 7 dpi (Fig 6J), suggesting that IFN-I production due to TLR7/9 signaling in CD103^+^ cDCs is important for normal levels of IFNγ production from both CD4^+^ and CD8^+^ T cells. Thus, altered T cell function and cytokine production may contribute to increased susceptibility to *Histoplasma* infection in TLR7/9^-/-^ mice.

**Fig 7:**
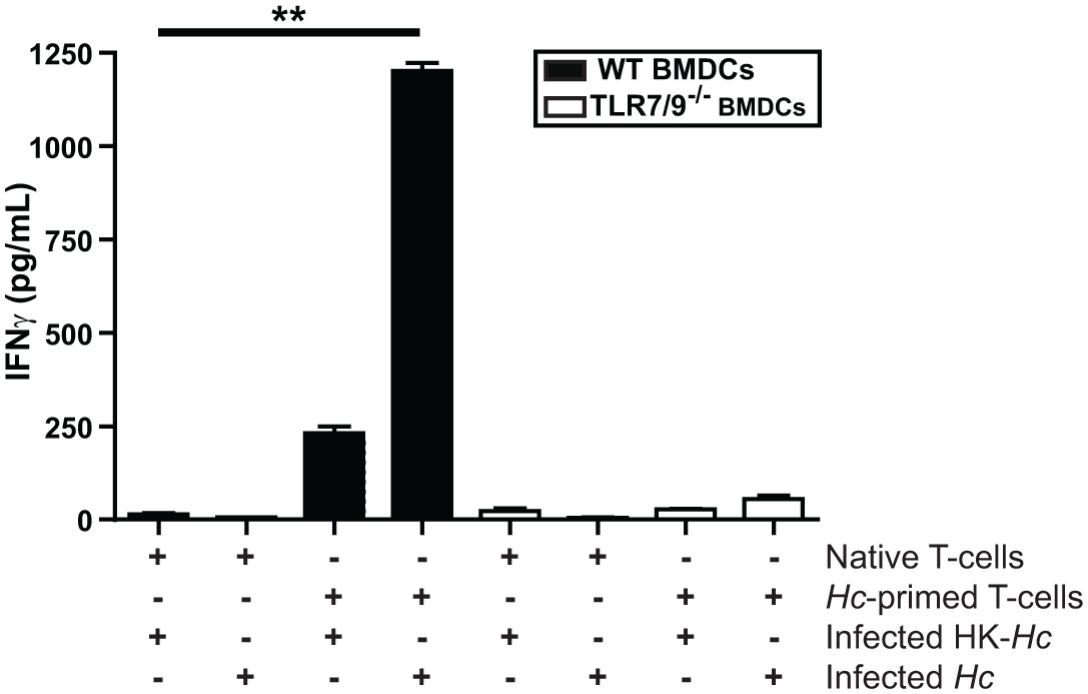
TLR7/9 is essential for proper IFNγ production during DC-T cell interactions. Wild-type and TLR7/9^-/-^ BMDCs were cocultured with heat-killed or live *Histoplasma capsulatum* (HK-*Hc* or *Hc*, respectively) at an MOI of 2. One hour post infection spleen-purified native or *Histoplasma*-primed CD4^+^ T cells were added to the BMDCs. Supernatants were collected at 48 hpi and IFNγ was quantified by ELISA. All error bars indicate SD. **p < 0.001; p values were determined by ANOVA.

Finally, we performed *in vitro* experiments to determine if wild-type and TLR7/9^-/-^ BMDCs differ in their ability to stimulate the production of IFNγ from CD4^+^ T cells, consistent with the defects observed *in vivo*. We purified both naïve and *Histoplasma*-primed CD4^+^ T cells from wild-type mouse spleens and cocultured them with wild-type or TLR7/9^-/-^ BMDCs. We monitored IFNγ levels in cell supernatants as a readout for T cell activation. IFNγ production was observed only in cocultures of *Histoplasma*-infected wild-type BMDCs with *Histoplasma*-primed CD4^+^ T cells. Notably, TLR7/9^-/-^ BMDCs were unable to activate primed CD4^+^ T cells (Fig 7). Taken together, these data uncover a critical role of TLR7/9 signaling in both the ability of CD103^+^ cDCs and alveolar macrophages to produce IFN-I during *Histoplasma* infection as well as TLR7/9-dependent instruction of the adaptive immune system to produce IFNγ and restrict fungal growth.

## Discussion

Endemic fungi such as *Histoplasma capsulatum* cause severe morbidity and mortality even in immunocompetent individuals [36] and the general increase in prevalence of fungal infections has stimulated interest in understanding the host response to these ubiquitous primary pathogens [37]. Here we demonstrate that TLR7/9 signaling in BMDCs is required to mount an IFN-I response, restrict intracellular *Histoplasma* proliferation, and maintain host cell viability. In contrast, BMMs do not induce IFN-I in response to infection with *Histoplasma* yeasts, and TLR7/9 do not influence the fate of infected macrophages. BMDCs that lack IFNAR are also unable to restrict fungal growth and are killed by *Histoplasma*, suggesting that IFN-responsive genes are required to control intracellular fungal proliferation. In the mouse model of *Histoplasma* infection, TLR7/9 are also required for IFN-I production and host survival. Mice lacking TLR7 and TLR9 display defects in production of IFNγ, a key cytokine for *Histoplasma* control, which likely contributes to their propensity to succumb to disease.

Previous observations indicate that whereas macrophages allow robust intracellular fungal growth and are susceptible to *Histoplasma*-mediated cell death, DCs are able to restrict *Histoplasma* growth and survive infection. We have shown that TLR7/9 signaling and the resultant IFN-I response are key for the fungistatic activity of DCs and the ability of these host cells to avoid pathogen-mediated death during *Histoplasma* infection. In addition, we found that TLR7/9 is required for proper DC-T cell interactions. We showed that BMDCs lacking TLR7/9 are no longer able to properly activate CD4^+^ T cells in vitro and observed a decrease in both IFNγ and IL12 production in vivo in the TLR7/9^-/-^ mice. Not surprisingly, TLR signaling in DCs is thought to have profound effects on DC function, such as enhancement of antigen capture and presentation, as well as modulation of protein translation and cell motility [38].

Interestingly, TLR7/9 activity requires that these receptors are processed in an acidic phagolysosome [39]. Previous studies have shown that *Histoplasma* resides in a non-acidified phagosome in macrophages [40], which suggests that TLR7/9 would not be competent to recognize *Histoplasma* ligands in these cells. This hypothesis is consistent with our observation that TLR7/9^-/-^ macrophages are indistinguishable from wild-type with respect to *Histoplasma* proliferation and host-cell death. Additionally, our previous work demonstrated that the ability of *Histoplasma* to maintain a neutral phagosome environment in macrophages is critical for intracellular fungal proliferation and macrophage death [41]. Experiments performed in our laboratory show that *Histoplasma* resides within an acidic compartment in BMDCs. Taken together, these data strongly suggest that pH-responsive host signaling pathways play a critical role in the differential ability of mammalian immune cells to succumb to or control *Histoplasma* infection.

We found that restriction of *Histoplasma* intracellular growth by BMDCs was clearly dependent on TLR7/9 and IFNAR, and addition of recombinant IFNβ was sufficient to restore host restriction to TLR7/9^-/-^ BMDCs. The basis of IFN-I-dependent restriction of *Histoplasma* by BMDCs is unknown, but it is well established that IFN-I trigger anti-microbial programs in immune cells via the induction of interferon-stimulated genes (ISGs) [4]. It may be that a subset of ISGs cause *Histoplasma* restriction in infected BMDCs.

TLR7 and/or TLR9 are essential for the induction of IFN-I in response to other fungal pathogens such as *Paracoccidioides brasiliensis*, *Candida albicans*, *Aspergillus fumigatus*, and *Cryptococcus neoformans* [5, 7, 9, 42–46], but little is known about which cells are responsible for producing IFN-I in vivo during infections with these fungi. Our *in vitro* differentiated BMDCs most closely resemble CD11b^+^ cDCs, and these cells were clearly capable of a robust IFN-I response to *Histoplasma* infection. Interestingly, during mouse infection with *Histoplasma*, we discovered that CD103^+^ cDCs, along with alveolar macrophages and CD11b+ cDCs, are the main IFN-I producing cells. Lung dendritic cells are thought to consist of three subsets: pDCs, CD11b^+^ cDCs, and CD103^+^ cDCs. Both types of cDCs have a distinct ontogeny from pDCs, and the differentiation of CD103^+^ cDCs in tissues is dependent on the transcription factor Batf3 [12]. The localization of pDCs, CD11b^+^ cDCs, and CD103^+^ cDCs in a resting lung is distinct: pDCs are distributed to conducting airways, parenchyma, and alveolar septa; CD11b^+^ cDCs are found in the lamina propria; and CD103^+^ cDCs are found in connective tissues where they extend processes into the alveolar space [47]. The differential role of these lung dendritic cell subsets in response to pathogens is an active area of research; for example, CD103^+^ cDCs are required to stimulate CD8^+^ T cell-mediated immunity in the context of infection with respiratory viruses such as influenza virus, whereas their role in the activation of CD4^+^ T cells is less clear [47]. Recently, CD103+ cDCs were shown to be critical in modulating the Th17 response to *A*. *fumigatus* infection [48]. Our work identifies a previously unknown role of CD103^+^ cDCs in type I interferon production in response to fungal infections.

Production of IFN-I is not limited to DCs, as illustrated by the ability of alveolar macrophages to produce IFN-I in response to *Histoplasma* infection. Indeed, previous work shows that IFN-I can be produced by different subclasses of immune cells, depending on the infectious trigger. pDCs are often reported as the main cell type that produces IFN-I during viral infection. In contrast, as we observed for *Histoplasma* infection, cell types other than pDCs produce IFN-I in response to distinct pathogens. For example, during infection with the parasite *Plasmodium chabaudi*, red pulp macrophages in the spleen produce IFN-I throughout infection [49]. In *Listeria monocytogenes* infection, a specialized subpopulation of TNF and iNOS producing DCs (Tip-DCs) are the main producers of IFN-I in the spleen and are essential for early infection containment [50]. It is interesting but not surprising that alveolar macrophages and BMMs differ in their ability to produce IFN-I in response to *Histoplasma* infection; BMMs are a powerful macrophage model but do not take on all characteristics of tissue macrophages. Additionally, we previously published that IFNAR^-/-^ mice showed decreased fungal burden during *Hc* infection [19], which contrasts with our observations here that IFNAR^-/-^ mice are more susceptible to *Hc* as determined by host survival. In our previous work, we did not perform survival studies, but simply examined organ colonization after a low-inoculum infection (2 x 10^4^ yeast cells). In the current study, we infected with 3 x 10^5^ yeast cells and examined survival without monitoring fungal burden. It may be the case that IFNAR^-/-^ mice exhibit decreased survival even in the face of decreased fungal burden, but further experiments are necessary to determine fungal burden and survival in a single cohort of wild-type and IFNAR^-/-^ mice in response to different inocula. These studies illustrate the complexity of the IFN-I response in the context of actual infections.

Unexpectedly, we found that mice lacking TLR7/9 accumulated *Histoplasma* cells in the brain, dispersed throughout the cortex and thalamus. In humans, central nervous system (CNS) involvement occurs in 2–20% of disseminated cases [51]. It has been reported that *Histoplasma* yeasts in the human brain appear to be extracellular [52], which was also the case for 50% of the *Histoplasma* in the TLR7/9^-/-^ mice. One intriguing idea is that *Histoplasma* is better able to survive within TLR7/9^-/-^ DCs compared to wild-type DCs, and that these host cells provide a vehicle or Trojan horse for the fungus to disseminate to the CNS. Understanding the mechanism of CNS colonization in TLR7/9^-/-^ mice may provide a useful model for studying microbial dissemination to the brain.

Finally, the ability of DCs to recognize and respond to a pathogen shapes the magnitude, duration, and character of the adaptive immune response [53]. We previously demonstrated the importance of MyD88 in a proper cytokine response and in host defense against *Histoplasma* [29]. Here we show that TLR7 and TLR9 are key in the initiation of a balanced cytokine response to *Histoplasma*. TLR7/9^-/-^ mice produce increased levels of IL6, TNF-α, and IL17A during *Histoplasma* infection, and similar results have been observed for the endemic fungal pathogen *P*. *brasiliensis* during infection of TLR9^-/-^ mice [46]. In particular, increased levels of IL17, which is associated with neutrophil recruitment [33], may be responsible for the high influx of neutrophils at 5 and 7 dpi in the lungs of TLR7/9^-/-^ mice infected with *Histoplasma*. Interestingly, approximately 50% of these neutrophils contained mCherry^+^ material, indicating that these cells had phagocytosed *Histoplasma*. Nonetheless, fungal burden remained uncontrolled in TLR7/9^-/-^ mice compared to wild-type. Collectively our data indicate the critical protective role of TLR7/9 signaling at both a cellular and organismal level during infection with a common fungal pathogen of humans.

## Materials and Methods

### Strains and culture conditions

*Histoplasma caspsulatum* strain G217B yeast (ATCC 26032) were grown in liquid *Histoplasma* macrophage media (HMM) or on HMM agarose plates [54]. Liquid cultures were grown in an orbital shaker at 37°C with 5% CO_2_. The mCherry+ *Histoplasma caspsulatum* (mCherry-*Hc*) strain was created by electroporating a P_CBP1_-mCherry-URA plasmid into the G217B *ura5Δ* strain, growing on HMM plates, and screening for the brightest colonies. Cells were inoculated from frozen stock onto HMM plates 3 weeks before each experiment. One week before infection, the strain was inoculated from solid media to liquid HMM and passaged 1:25 every three days and then diluted the night before an infection to reach mid-logarithmic-phase at the time of infection. In preparation for infection of both mice and *in vitro* cell cultures, fungal cells were washed once with PBS, sonicated briefly using a Fisher Scientific Sonic Dismembrator Model 100 (5 seconds on setting 2), centrifuged for 3 min at 500 RPM to remove large clumps, and enumerated using a hemacytometer.

### Mice

Female C57Bl/6J mice (strain 000664) were originally purchased from Jackson Laboratory. All knockout mice were in the C57Bl/6J background. IFNAR^-/-^ and TLR7^-/-^ mice were a gift from Mehrdad Matloubian (UCSF), TRIF^-/-^ and TLR3^-/-^ mice were a gift from Anthony DeFranco (UCSF), and *mob* mice (IFNβ/YFP) were a gift from Charlie Kim (UCSF). Batf3^-/-^ mice were purchased from Jackson Laboratory. MyD88^-/-^ mice were originally purchased from Jackson Laboratory and then bred in a barrier facility at UCSF. Strain information is as follows: IFNAR^-/-^ (strain 002509; B6.129S2-Ifnar1tm1Agt/Mmjax), MyD88^-/-^ (strain 009088; B6.129P2(SJL)-Myd88^tm1.1Defr/^J), TRIF^-/-^ (strain 005037; C57BL/6J-Ticam1Lps2/J), TLR3^-/-^ (strain 009675; B6N.129S1-Tlr3tm1Flv/J), TLR7^-/-^ (strain 008380; B6.129S1-Tlr7tm1Flv/J), TLR9^-/-^ (strain 014534; C57BL/6J-Tlr9M7Btlr/Mmjax), *mob* (strain 010818; B6.129-Ifnb1tm1Lky/J), and Batf3^-/-^ (strain 013755; B6.129S(C)-Batf3tm1Kmm/J). The double TLR7 and 9 knockout mice (TLR7/9^-/-^) were generated in the laboratory of Charlie Kim (UCSF). To generate the *mob* TLR7/9^-/-^ mice, we crossed *mob* TLR7^-/-^ and *mob* TLR9^-/-^ parents, which were a kind gift from Charlie Kim (UCSF).

### Ethics statement

All mouse experiments were performed in compliance with the National Institutes of Health *Guide for the Care and Use of Laboratory Animals* and were approved by the Institutional Animal Care and Use Committee at the University of California San Francisco (protocol AN099634-02B).

### Macrophage and dendritic cell differentiation and infections

Bone marrow macrophages (BMMs) and bone marrow dendritic cells (BMDCs) were cultured in parallel from the same bone marrow for all experiments. Briefly, femurs were isolated from 8–10 week old female C57Bl/6J mice or mutant mice (IFNAR^-/-^, MyD88^-/-^, TRIF^-/-^, TLR3^-/-^, TLR7^-/-^, TLR9^-/-^, or TLR7/9^-/-^). The bone marrow was extracted and red blood cells lysed with ACK lysing buffer. For BMMs, cells were plated in 10 cm petri dishes and differentiated for 7 days with one media addition of Dulbecco’s Modified Eagle Medium, D-MEM High Glucose (UCSF Cell Culture Facility), 20% heat-inactivated Fetal Bovine Serum (FBS; Hyclone), 10% v/v CMG supernatant (the source of CSF-1), 2 mM glutamax, 110 μg/mL sodium pyruvate (UCSF Cell Culture Facility), and penicillin and streptomycin (UCSF Cell Culture Facility). Adherent cells were scraped and plated 24 hours pre-infection. For BMDCs, cells were plated in 24-well plates and differentiated for 6 days with one media addition in RPMI-1640, 10% heat-inactivated Fetal Bovine Serum (FBS; Hyclone), mIL4 (20 ng/mL; Gibco), mGM-CSF (10 ng/ml; PeproTech), 2 mM glutamax, 110 μg/mL sodium pyruvate (UCSF Cell Culture Facility), and penicillin and streptomycin (UCSF Cell Culture Facility). Non-adherent cells were collected and purified on a CD11c column and allowed to rest for 48 hours post-purification to reduce column-induced activation, which was monitored with activation markers (CD68 and MHC II) via flow cytometry. Ultra pure lipopolysaccharide (LPS) was purchased from InvivoGen. Recombinant murine IFN-β was obtained from EMD Millipore.

### BMDC-T cell co-cultures

To isolate CD4^+^ T cells, mice were either mock-infected or infected with a sub-lethal dose (1x10^4^) of wild-type *Histoplasma*. At 14 dpi, spleens were dissociated in HBSS using the GentleMACS Dissociator (Miltenyi Biotec) and leukocytes were enriched via Lympholyte-M separation (Cedarlane). B cells were depleted by incubating the cells on anti-IgM and anti-IgG antibody-coated plates for 2 h at 37°C. Non-adherent cells were collected and T cells were purified by negative selection using a Pan-T cell Isolation Kit (Miltenyi Biotec), followed by positive selection using anti-mouse CD4 microbeads (Miltenyi Biotec). CD4^+^ T cells were allowed to rest for 24 h before co-culture. Purified wild-type and TLR7/9^-/-^ BMDCs were seeded in 96-well plates at a density of 3x10^4^ cells/well and cocultured with live or heat-killed *Histoplasma* at an MOI of 2. One hour post infection, 1x10^5^ of either native or *Histoplasma*-primed purified CD4^+^ T cells were added. Medium was removed 48 hpi and IFNγ was quantified using a Ready-Set-Go! ELISA kit (eBioscience).

### Cytotoxicity assay

In 24-well tissue culture treated dishes, 2x10^5^ BMDCs or BMMs were infected, in duplicate, with *Histoplasma* strains at an MOI of 2. Cytotoxic assays were performed as previously described [41]. Briefly, LDH levels in the infected BMDCs or BMMs supernatants were measured to monitor host cell lysis. LDH solution was made from equal volumes of the following solutions: (1) 2 mg/ml IodoNitroTetrazolium chloride (INT) in PBS, (2) 36 mg/ml Lithium L-lactate in 10 mM Tris, pH 8.5, and (3) 1x NAD+/diaphorase in PBS containing 1% BSA (diluted from a 10X stock solution (13.5 U/mL diaphorase, 3 mg/mL NAD+, 0.03% BSA, and 1.2% sucrose in PBS). 30 μl of supernatant was added to 30 μl of LDH solution and then incubated for 30 minutes in the dark. 40 μl of 1 M acetic acid was added to stop the reaction. The OD_490_ was then measured using the Molecular Devices Spectramax Plus 384 plate reader. The percentage BMM lysis at each time-point was calculated as the percentage of the total LDH from uninfected cells lysed in 1% Triton X-100 (in DMEM without phenol red) at 2 hpi. Due to continued replication of host cells over the course of the experiment, the total LDH at later time points is greater than the total LDH from the 2hr time point, resulting in an apparent % lysis that is greater than 100%.

### Intracellular replication assay

In 24-well tissue culture treated dishes, 2x10^5^ BMDCs or BMMs were infected, in duplicate, with *Histoplasma* at an MOI of 2. Phagocytosis rates of all BMDCs (IFNAR^-/-^, TLR3^-/-^, TLR7^-/-^, TLR9^-/-^, and TLR7/9^-/-^) were equivalent as calculated by flow cytometric analysis of BMDCs exposed to mCherry+ *Histoplasma* cells. At various time points post-infection, the media was removed from each well and 1 ml ddH_2_O was added. After a 5 min incubation, the host cells were mechanically lysed by vigorous pipetting. The lysate was collected, sonicated, diluted in HMM and plated for *Histoplasma* colony forming units (CFUs) on HMM-agarose plates at 37°C. Colonies were counted 10 days later. The relative CFU at time x was calculated as (CFU_tx_)/(CFU_t0_).

### Microarray hybridizations and analysis

In 6-well tissue culture treated dishes, 1x10^6^ BMDCs or BMMs were subjected to either cocultured with UV-treated or live *Histoplasma* yeasts at an MOI of 4. UV-treated (UVT) yeasts were prepared by subjecting *Histoplasma* to UV light (UV Stratalinker 1800) for 1 hour; these yeasts failed to generate colonies when plated on HMM agarose. Total RNA was isolated at various time points post-infection using the RNeasy Mini Extraction Kit (Qiagen) and amplified using the Amino Allyl MessageAmp II aRNA Amplification Kit (Applied Biosystems). Microarray probes were generated by coupling aRNA to Cy5 or Cy3 monofunctional dyes (Amersham). Probes were hybridized to MEEBO (mouse exonic evidence-based oligonucleotide) microarrays (http://alizadehlab.stanford.edu/) using a pooled reference strategy in which Cy5-labeled aRNA samples corresponding to each time point were competitively hybridized against a Cy-3 labeled reference pool containing equal amounts of all infected and uninfected samples. Arrays were hybridized for 36 hours at 63°C and scanned on a GenePix 4000B scanner (Axon Instruments). Arrays were gridded using Spotreader (Niles Scientific) and results files were generated using GenePix Pro, version 6.0 (Molecular Devices). Poor quality features as identified by visual inspection were flagged and excluded from further analysis. Ratio of medians (635/532) were extracted for each array feature, excluding features for which the sum of medians for the 635 nm and 532 nm channels was > = 500 intensity units. To extract relative gene expression ratios, these data were transformed relative to the mock-infected samples. Raw microarray data are available at the Gene Expression Omnibus (GEO) databases under GEO series accession number GSE70505.

### Type I IFN reporter assay

BMMs and BMDCs were plated at 5x10^5^ per well on 6-well plates and infected in triplicate with 2x10^6^ *Histoplasma* yeast. At various time points post-infection, 500 μl of culture media was taken and flash frozen in liquid nitrogen and stored at -80°C. The amount of IFN-I secreted by these cells during *Histoplasma* infection was detected with the ISRE-L929 luciferase reporter cell line [24]. ISRE-L929 cells were plated at 5x10^4^ per well in a 96-well white opaque plate and incubated with 100 μl of supernatant for 5–8 h. Cells were then lysed and luciferase activity was detected by using Bright Glow Assay (Promega, E-2620) and light emission measurement by luminescence counter (VICTOR2, PerkinElmer).

### Mouse infections

Eight-to-ten week old female mice were anesthetized with isoflurane and infected intranasally with *Histoplasma* yeasts. In preparation for infection, mid-logarithmic cultures of *Histoplasma* were washed once with PBS, sonicated for 5 seconds, centrifuged at 500 rpm for 3 min to remove large clumps, and counted by hemacytometer to determine cell concentration. To monitor mouse survival, ten mice were infected intranasally with 3x10^5^ yeast in approximately 25 μl PBS. At 4 hpi, the lungs and spleens were harvested and homogenized from 3 infected mice. These homogenates were plated for *Histoplasma* CFUs on Brain-Heart Infusion agar (BHI) plates and incubated at 30°C. The remaining mice were monitored daily for symptoms of disease (i.e. weight loss, lack of activity/response to stimulus, panting, lack of grooming). Mice were sacrificed after they exhibited 3 days of sustained weight loss greater than 25% of their maximum weight in conjunction with one other symptom of disease. For the in vivo colonization assay, mice were infected with 3x10^5^ *Histoplasma* yeasts. For each time point, lungs, spleens, and brains were harvested from 5 infected mice. These organs were homogenized and CFUs were enumerated on BHI plates at 30°C.

### Histology

Intact brains were placed directly into fixative (10% formaldehyde in 0.1M phosphate buffer with 0.9% saline). After a minimum of 24h, they were transferred to cryoprotectant (30% sucrose in 0.1M phosphate buffer with 0.9% saline) at 4°C until tissues sank. Brains were embedded in Tissue-Tek and 25μm sections were cut on a freezing microtome. They were then mounted onto gelatinized slides and allowed to air-dry overnight. Coverslips were mounted using Fluoro-gel II with DAPI (Electron Microsopy Sciences). Sections were viewed on a Zeiss Axiovert 200 inverted microscope. Images were taken using a Zeiss Axiocam MRm camera.

### Isolation of lungs, spleens and brains

For CFU and cytokine analysis on mouse lungs and spleens, the organs were isolated, homogenized on ice with 1x cOmplete protease inhibitor (Roche) and flash frozen until analysis. For flow cytometry analysis, lungs, spleens and brains were perfused with PBS and dissociated in Hanks Buffered Salt Solution (HBSS) containing 80 U/mL DNase; D4527 (Sigma). Lungs and spleens were treated with 0.75U/mL Liberase TM (Roche) using a GentleMACS Tissue Dissociator (Miltenyi Biotec). Red blood cells were hypotonically lysed with ACK lysis buffer and the remaining cells filtered through a 70 μM cell strainer (Becton Dickenson). Brains were manually dissociated between two glass cover slips and cells were washed before counting.

### Cytokine analysis

Lung and spleen homogenates were centrifuged at 4°C and the supernatant was sterilized using a 0.2 μm Co-Star-X centrifuge tube (Corning). Mouse Cytometric Bead Array Flex Sets (Becton Dickenson) were used according to the manufacturer’s instructions to determine concentrations (pg/mL) of IL6, IFN-γ, IL17a, IL1β, and TNFα.

### Flow cytometry

2x10^6^ lung or spleen cells were resuspended in PBS and stained with Fixable Viability Dye eFluor 450 (eBiosciences) for 20 minutes, then washed and resuspended in PBS containing 1% heat-inactivated FBS, 1 mM EDTA, 10 μg/mL CD16/32 and 0.1% sodium azide. Cells were stained for 30 minutes with appropriate antibodies, fixed in BD Stabilizing Fix, and stored at 4°C until analysis on an LSR II (Becton Dickenson). Antibodies used to identify monocytes, macrophages, neutrophils, and DCs were as follows: neutrophils (CD11c^-^CD11b^+^SiglecF^lo^Ly6G^+^), monocytes (CD11c^-^CD11b^+^MHCII^-^CD64^+^), CD103^+^ cDCs (MHCII^+^CD11c^+^CD11b^-^CD24^+^CD103^+^), CD11b^+^ cDCs (MHCII^+^CD11c^+^CD11b^+^CD103^-^), and pDCs (CD11c^+/-^CD11b^-^CD103^-^B220^+^). APC-CD45, PE-Cy7-B220, and APC.780-MHCII (eBioscience), Alexa700-CD11c and PE-Cy7-SiglecF (Miltenyi Biotec), BV510-CD103, BV510-Ly6G, BV605-CD11b and PE-CD24 (Becton Dickenson), and BV711-CD64 and BV421-CD19 (BioLegend). The antibodies used to identify T cells and NK cells were as follows: APC-CD4, PerCp-Cy5.5-CD8, PE-Cy7-CD69, and PE-CD69 (Becton Dickenson) and PerCp-Cy5.5-NK1.1, FITC-CD3ε, and APC-gamma delta T cell receptor (γδ TCR) (eBiosciences). Flow cytometry data was analyzed using FlowJo version 7.6.5.

### Plasmacytoid dendritic cell depletion

To deplete the pDCs fraction, mice were treated with 100 μg anti-pDCA-1 mAb functional grade (Miltenyi Biotec) or 100 μg control Ab (rat IgG; Sigma-Aldrich) injected i.p. in 100 μL PBS 1 day before infection and 1, 3, 5, and 6 dpi. The efficiency of the depletion was controlled by harvesting cells isolated from the spleen and monitoring CD11c, CD11b, B220 and SiglecH populations by subsequent flow cytometry analysis. The percentage of pDCs in spleen (gated on a CD11c^+/-^SiglecH^+^B220^+^CD11b^-^ fraction) was measured 7 dpi after treatment with normal rat IgG or anti-pDCA-1 mAb.

### Statistical analysis

Statistical analysis for experiments was performed using Prism (GraphPad Software, San Diego, CA). Analysis of variance (ANOVA) with Tukey’s post-test was used to analyze significance of cytokine expression and flow cytometry experiments. The log rank sum test was used to analyze survival. Statistically significant differences were denoted for p values *p< 0.05 or **p<0.001.

## Supporting Information

S1 Fig Flow cytometry analysis of purified BMDCs. Bone marrow was cultured with GM-CSF ± IL4. At day 6, CD11c^+^ cells were harvested after purification with a CD11c column. The surface expression of CD11c, CD11b, MHCII, CD135, CD115, CD103, and SiglecH was analyzed by flow cytometry. Boxes depict gates and numbers correspond to percentage of cells in each gate. Data are representative of two experiments.
(TIF)

S2 Fig Dendritic cells restrict *Histoplasma* growth. BMMs or BMDCs were infected with live *Histoplasma* yeasts at an MOI of 4. Host cells were osmotically lysed and CFUs representing intracellular yeast cells were enumerated. Representative experiment of 3 replicates is shown.
(TIF)

S3 Fig IFN-I production and *Histoplasma* restriction in DCs is dependent on TLR7 and TLR9 signaling. **(A)** WT, TLR3^-/-^, TLR7^-/-^, or TLR9^-/-^ BMDCs were infected with *Histoplasma* yeasts at an MOI of 4 and IFN-I was measured at 12 hpi. WT, TLR7^-/-^, or TLR9^-/-^ BMM and BMDCs were either mock-infected or infected with *Histoplasma* yeasts at an MOI of 2 and monitored for **(B, D)** host-cell lysis via LDH activity and **(C, E)** CFUs. Representative experiment of 3 replicates is shown and error bars indicate SD. **p<0.001; p values were determined by ANOVA.
(TIF)

S4 Fig *Histoplasma* in the brain is mainly extracellular. TLR7/9^-/-^ mice were intranasally infected with a sublethal dose of 3x10^5^ mCherry-*Hc* yeasts. **(A)** Kaplan-Meir survival curves of female WT (n = 10), TLR7/9^-/-^ (n = 10) or PBS-treated (uninfected) (n = 4) mice. Lungs, spleens and brains of infected WT and TLR7/9^-/-^ mice were harvested, homogenized and plated for CFUs at the indicated days post-infection (dpi) (n = 5 mice/time-point). **(B)** 14 dpi brains were collected. Percentage of mCherry positive CD11c^+^ DCs, microglia, and extracellular yeasts. Each symbol represents a single mouse. All results are representative of at least three experiments. **(C)** Histology section of mCherry-*Hc* (indicated by arrow) in the choroid plexus of the brain. *p<0.05; **p<0.001; p values were determined by ANOVA.
(TIF)

S5 Fig Flow cytometry of inflammatory cell types in the mouse lungs after *Histoplasma* infection. The basic set-up for all downstream analysis included the following: live cells were first selected based on negative staining of Live/Dead stain, then singlets were selected and debris was removed. Subsequently, CD45 positive cells were selected. Alveolar macrophage gate based on CD11c^+^CD11b^-^SiglecF^Hi^CD64^+^. Neutrophil gate based on CD11c^-^CD11b^+^SiglecF^lo^Ly6G^+^. Monocyte gate based on CD11c^-^CD11b^+^MHCII^-^CD64^+^. CD103^+^ cDC gate based on MHCII^+^CD11c^+^CD11b^-^CD24^+^CD103^+^ and CD11b^+^ cDC gate based on MHCII^+^CD11c^+^CD11b^+^CD103^-^. Plasmacytoid DC (pDC) gate based on CD11c^+/-^CD11b^-^CD103^-^B220^+^. Numbers shown represent the percentage of cells within the gates.
(TIF)
