# Supplementary figures and images for: CD103+ Conventional Dendritic Cells Are Critical for TLR7/9-Dependent Host Defense against Histoplasma capsulatum, an Endemic Fungal Pathogen of Humans
Source: PLoS Pathog. 2016 Jul 26;12(7):e1005749. doi: 10.1371/journal.ppat.1005749 (PMC4961300; doi:10.1371/journal.ppat.1005749)

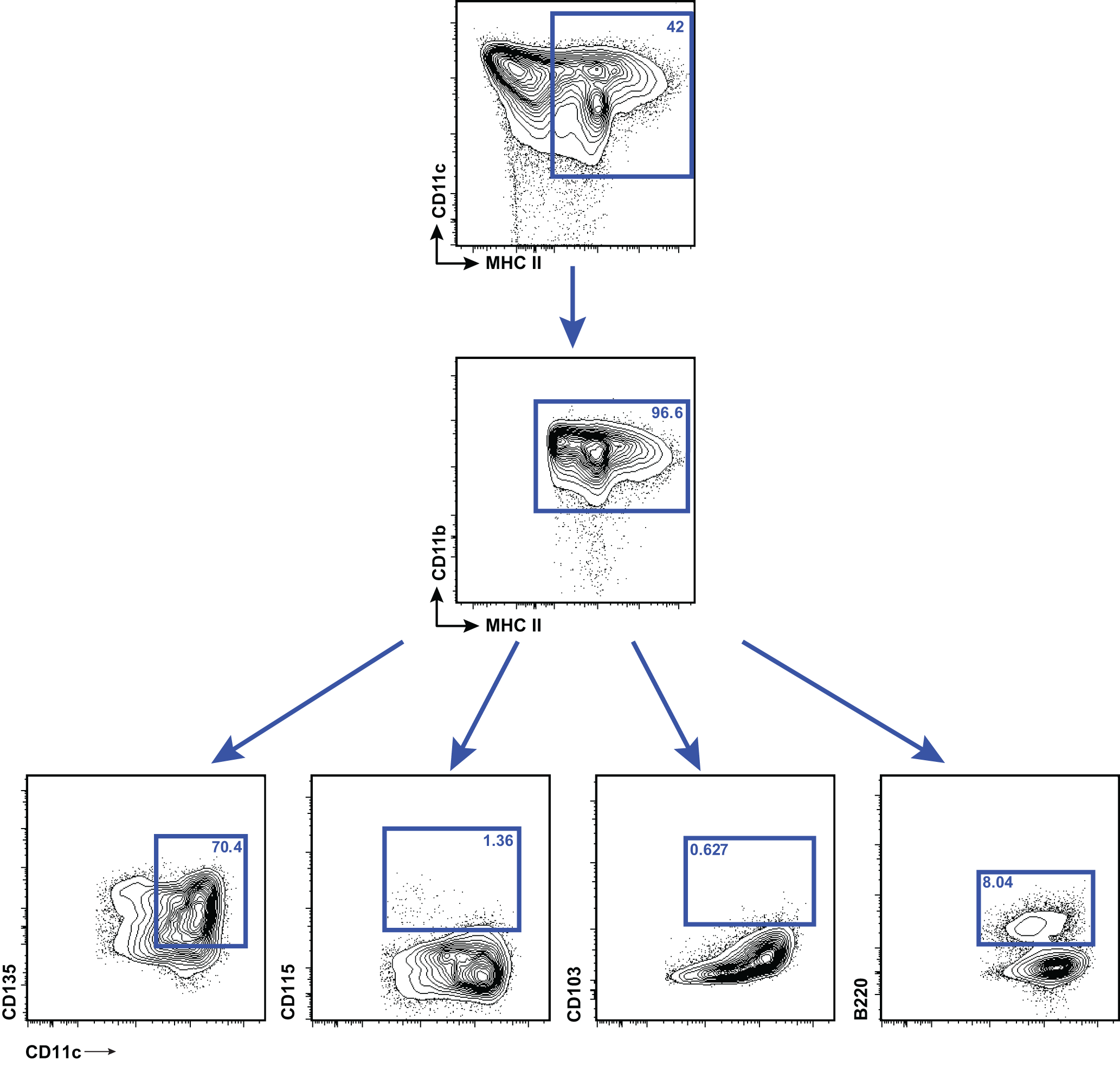

Supplement: S1 Fig — Bone marrow was cultured with GM-CSF ± IL4. At day 6, CD11c+ cells were harvested after purification with a CD11c column. The surface expression of CD11c, CD11b, MHCII, CD135, CD115, CD103, and SiglecH was analyzed by flow cytometry. Boxes depict gates and numbers correspond to percentage of cells in each gate. Data are representative of two experiments. (TIF) [file ppat.1005749.s001.tif]

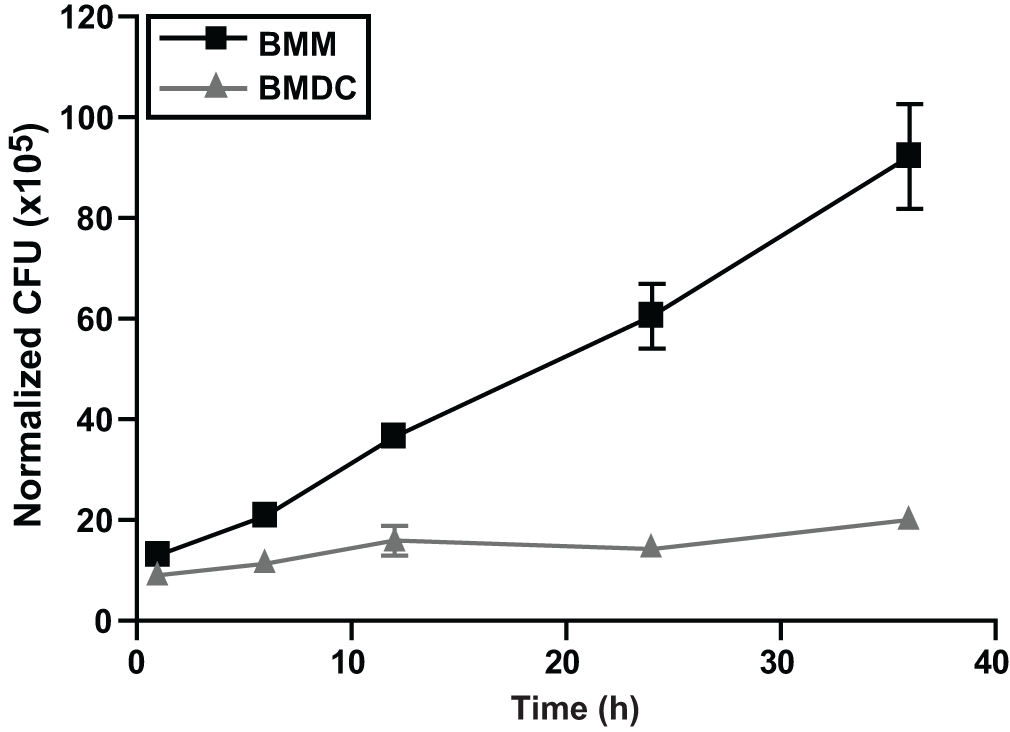

Supplement: S2 Fig — BMMs or BMDCs were infected with live Histoplasma yeasts at an MOI of 4. Host cells were osmotically lysed and CFUs representing intracellular yeast cells were enumerated. Representative experiment of 3 replicates is shown. (TIF) [file ppat.1005749.s002.tif]

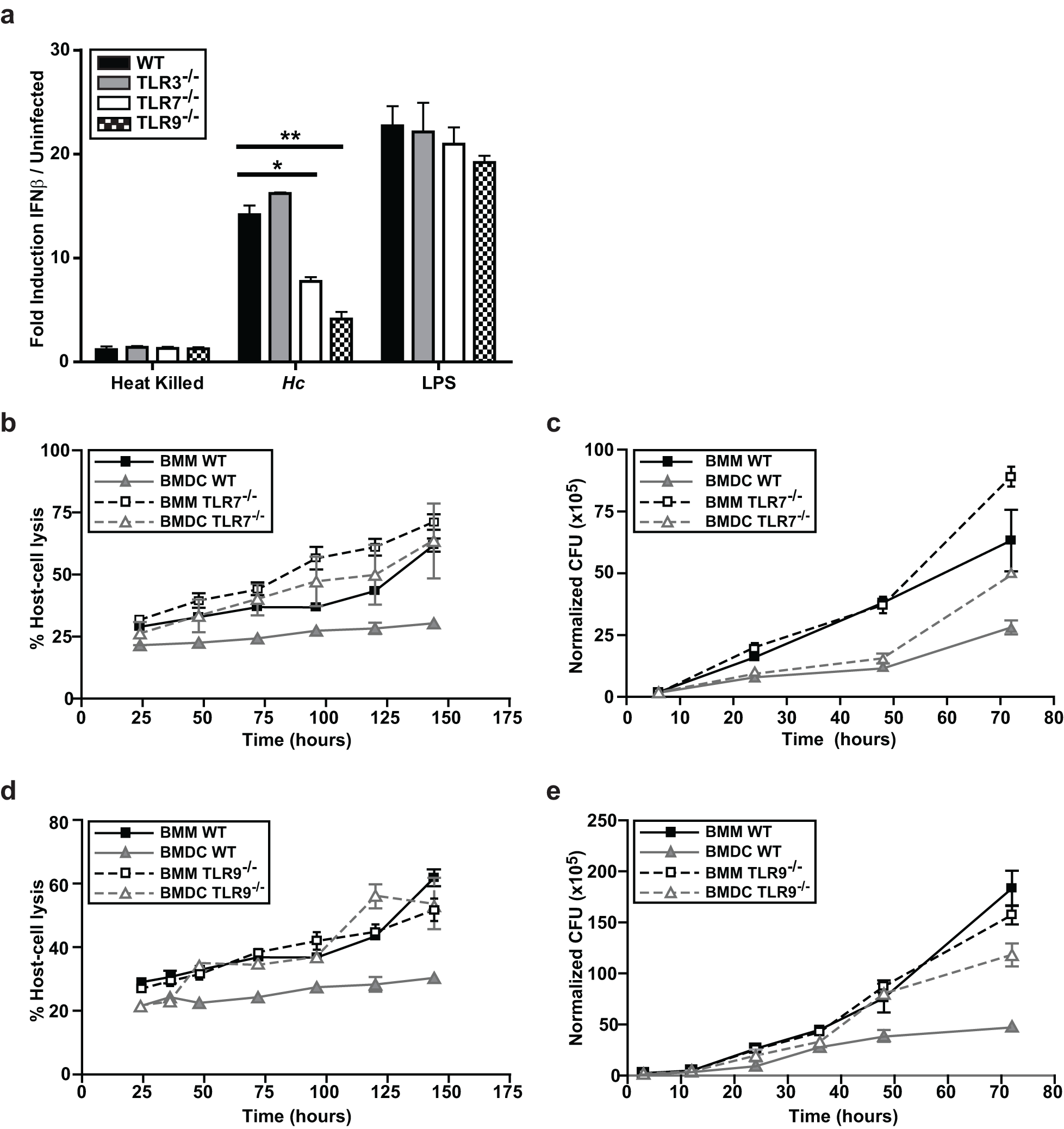

Supplement: S3 Fig — (A) WT, TLR3-/-, TLR7-/-, or TLR9-/- BMDCs were infected with Histoplasma yeasts at an MOI of 4 and IFN-I was measured at 12 hpi. WT, TLR7-/-, or TLR9-/- BMM and BMDCs were either mock-infected or infected with Histoplasma yeasts at an MOI of 2 and monitored for (B, D) host-cell lysis via LDH activity and (C, E) CFUs. Representative experiment of 3 replicates is shown and error bars indicate SD. **p<0.001; p values were determined by ANOVA. (TIF) [file ppat.1005749.s003.tif]

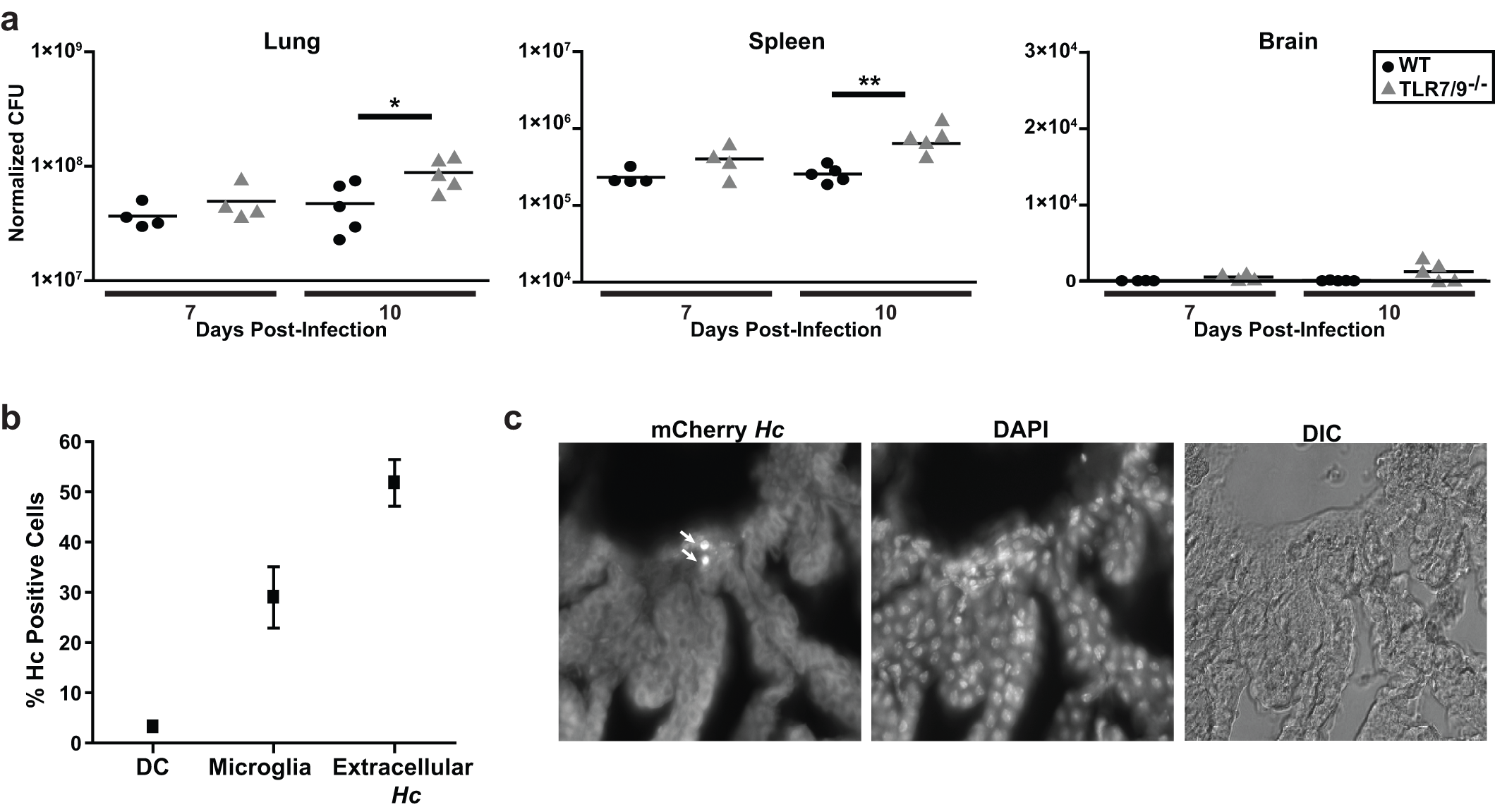

Supplement: S4 Fig — TLR7/9-/- mice were intranasally infected with a sublethal dose of 3x10^5^ mCherry-Hc yeasts. (A) Kaplan-Meir survival curves of female WT (n = 10), TLR7/9-/- (n = 10) or PBS-treated (uninfected) (n = 4) mice. Lungs, spleens and brains of infected WT and TLR7/9-/- mice were harvested, homogenized and plated for CFUs at the indicated days post-infection (dpi) (n = 5 mice/time-point). (B) 14 dpi brains were collected. Percentage of mCherry positive CD11c+ DCs, microglia, and extracellular yeasts. Each symbol represents a single mouse. All results are representative of at least three experiments. (C) Histology section of mCherry-Hc (indicated by arrow) in the choroid plexus of the brain. *p<0.05; **p<0.001; p values were determined by ANOVA. (TIF) [file ppat.1005749.s004.tif]

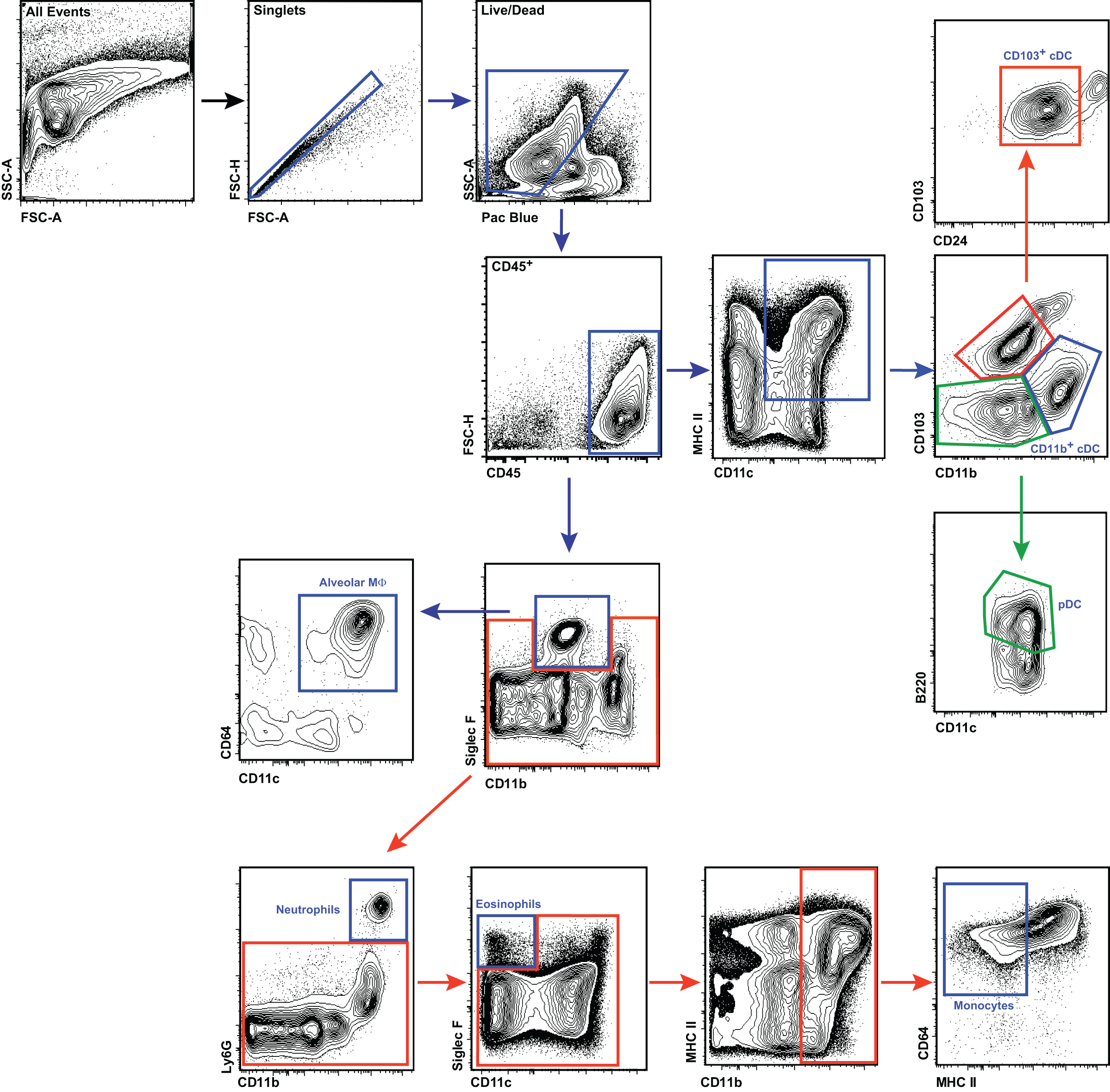

Supplement: S5 Fig — The basic set-up for all downstream analysis included the following: live cells were first selected based on negative staining of Live/Dead stain, then singlets were selected and debris was removed. Subsequently, CD45 positive cells were selected. Alveolar macrophage gate based on CD11c+CD11b-SiglecFHiCD64+. Neutrophil gate based on CD11c-CD11b+SiglecFloLy6G+. Monocyte gate based on CD11c-CD11b+MHCII-CD64+. CD103+ cDC gate based on MHCII+CD11c+CD11b-CD24+CD103+ and CD11b+ cDC gate based on MHCII+CD11c+CD11b+CD103-. Plasmacytoid DC (pDC) gate based on CD11c+/-CD11b-CD103-B220+. Numbers shown represent the percentage of cells within the gates. (TIF) [file ppat.1005749.s005.tif]
